# Chemokines in Severe Cutaneous Adverse Reactions (SCARs)

**DOI:** 10.3390/biom11060847

**Published:** 2021-06-06

**Authors:** Fumi Miyagawa, Hideo Asada

**Affiliations:** Department of Dermatology, Nara Medical University School of Medicine, 840 Shijo, Kashihara, Nara 634-8522, Japan; asadah@naramed-u.ac.jp

**Keywords:** severe cutaneous adverse reactions, Stevens-Johnson syndrome, toxic epidermal necrolysis, drug reaction with eosinophilia and systemic symptoms/drug-induced hypersensitivity syndrome, chemokine, HHV-6, chemokine mimicry

## Abstract

Although the incidence of severe cutaneous adverse reactions (SCARs) to medications is very low, SCARs can result in disability or even death if they are not diagnosed and treated properly. As the rapid recognition of SCARs is essential, it is necessary to develop diagnostic markers for them that can also be used to assess severity and predict outcomes in the early phase. In addition, it is important to identify novel therapeutic targets for SCARs. Chemokines are chemotactic cytokines that control the migratory patterns and locations of immune cells and usually exhibit markedly specific associations with certain human diseases. In Stevens-Johnson syndrome (SJS)/toxic epidermal necrolysis (TEN), the Th1-associated chemokines chemokine (C-X-C motif) ligand 9 (CXCL9) and CXCL10 predominate, while in drug-induced hypersensitivity syndrome (DIHS)/drug reaction with eosinophilia and systemic symptoms (DRESS), the levels of the Th2-associated chemokines chemokine (C-C motif) ligand 17 (CCL17) and CCL22 are markedly elevated. We suggest that the distinct chemokine profiles of SJS/TEN and DIHS/DRESS can be used to aid their differential diagnosis. CXCL10 has also been reported to be associated with the development of long-term sequelae in DIHS/DRESS. This review focuses on the chemokines involved in the pathogenesis and adjuvant diagnosis of SCARs, particularly SJS/TEN and DIHS/DRESS, but also provides a brief overview of SCARs and the chemokine superfamily. As it is being increasingly recognized that an association exists between human herpesvirus 6 (HHV-6) and DIHS/DRESS, the possible roles of the chemokine/chemokine receptor homologs encoded by HHV-6 in the pathogenesis of DIHS/DRESS are also discussed.

## 1. Introduction

Severe cutaneous adverse reactions (SCARs) are a rare heterogeneous group of delayed hypersensitivity reactions, mainly drug-induced reactions, which are associated with significant morbidity and mortality [1]. SCARs include Stevens-Johnson syndrome (SJS), toxic epidermal necrolysis (TEN), drug-induced hypersensitivity syndrome (DIHS)/drug reaction with eosinophilia and systemic symptoms (DRESS), and acute generalized exanthematous pustulosis (AGEP) [2]. SJS and TEN belong to the same entity lineage; i.e., they affect the skin and mucous membranes and are characterized by cutaneous erythema with blister formation and hemorrhagic erosive lesions of the mucous membranes [3]. The typical presentation of DIHS/DRESS involves a rash, fever, hematological abnormalities (leukocytosis, eosinophilia, and/or atypical lymphocytosis), the involvement of multiple visceral organs, sequential herpesvirus reactivation, and a prolonged clinical course (often due to relapses) [4]. On the other hand, AGEP typically involves the sudden appearance of dozens of nonfollicular pustules on a background of diffuse erythema, predominantly in the folds of the skin [5]. Although our understanding of many aspects of SCARs has evolved considerably, the prognosis of SCARs, especially that of SJS/TEN and DIHS/DRESS, still remains poor. In this review, we discuss the potential use of chemokines for diagnosing SCARs early and improving their outcomes. Chemokines are a family of chemotactic cytokines, which play vital roles in cell migration from the blood into tissue through venules and vice versa, and in the induction of cell movement in response to a chemical (chemokine) gradient by a process known as chemotaxis. Although they were initially identified as leukocyte attractants and important mediators of acute inflammation, we now know that they also contribute to many aspects of immunity [6,7]. These critical roles of chemokines in inflammation/immune responses mean that they may be useful for diagnosing SCARs. This review will also provide a brief overview of the chemokine superfamily and the roles of chemokines in T-helper (Th) cell differentiation. We also discuss the possible roles of viral chemokines/chemokine receptors in the pathogenesis of DIHS/DRESS.

## 2. Clinical Characteristics of SCARs

### 2.1. SJS/TEN

SJS and TEN are two related life-threatening mucocutaneous disorders within a continuous spectrum [2,3]. Both are characterized by the acute onset of erythema and blisters/erosive lesions due to necrotic damage to the epidermis, and hemorrhagic and erosive lesions of the mucous membranes. Frequently, fever and malaise are the first symptoms of SJS/TEN [3]. Widespread erythematous or purpuric macules (spots) or flat atypical targets, predominantly on the trunk, are the characteristic cutaneous manifestations of SJS [8,9]. In TEN, widespread macules and flat atypical targets precede epidermal sloughing in most cases (TEN with spots), although in a few cases TEN develops on large erythematous areas without any purpuric macules or targets and skin detachment is seen in little more than 10% of the body surface area (BSA) (TEN without spots) [8,9]. Roujeau’s group classified cases in which skin detachment was seen on <10% of the BSA as SJS, those in which skin detachment was seen on >30% of the BSA as TEN, and those in which skin detachment was seen on between 10% and 30% of the BSA as SJS-TEN overlap [8,9]. In contrast, in Japan, SJS is defined when skin detachment affects <10% of the BSA, while TEN involves detachment of ≥10% of the BSA [10].

Large epidemiological investigations of SJS/TEN patients conducted by the RegiSCAR (International Registry of Severe Cutaneous Adverse Reactions to drugs) group demonstrated that the mortality rate of SJS/TEN was 23% at 6 weeks and 34% at 1 year [11]. Studies conducted in the United States demonstrated that the mean estimated incidences of SJS, SJS-TEN overlap, and TEN were 9.2, 1.6, and 1.9 per million adults per year, respectively, and the mean adjusted mortality rate was 4.8% for SJS, 19.4% for SJS-TEN overlap, and 14.8% for TEN [12]. Likewise, a nationwide epidemiological survey conducted in Japan showed that the annual prevalence per million was 2.5 for SJS and 1 for TEN, and the mortality rate was 4.1% for SJS and 29.9% for TEN [10].

### 2.2. DIHS/DRESS

DIHS/DRESS is a potentially fatal multiorgan hypersensitivity reaction that appears after prolonged exposure to certain drugs [13,14]. These include carbamazepine, phenytoin, phenobarbital, zonisamide, lamotrigine, allopurinol, sulfasalazine, dapsone, mexiletine, minocycline, and vancomycin [15,16]. The reactivation of human herpesvirus 6 (HHV-6) has been implicated in cases of DIHS/DRESS that occur 2–3 weeks after the onset of a rash [17,18,19]. DIHS/DRESS is characterized by a delayed onset, occurring 3 weeks to 3 months after the start of the causative drug, and the deterioration of clinical symptoms after the discontinuation of the causative drug [13,14,15]. The symptoms of DIHS/DRESS include fever, lymphadenopathy, and rash development, which typically begins with maculopapular eruptions and often generalizes into severe exfoliative dermatitis or erythroderma. Facial and periorbital edema with pinhead-sized pustules is one of the characteristic features of DIHS/DRESS [15]. The laboratory findings of DIHS/DRESS include leukocytosis, eosinophilia, atypical lymphocytosis, and liver abnormalities to varying extents [15]. Systemic involvement typically manifests as hepatitis, but may also manifest as interstitial pneumonitis, interstitial nephritis, and/or myocarditis [16]. DIHS/DRESS can be associated with cytomegalovirus (CMV) reactivation, which occurs 3–7 weeks after onset and can lead to fatal outcomes if the patient develops overt CMV disease [20,21]. Some of the complications of DIHS/DRESS, such as pneumonia, hepatitis, and gastroenteritis, are known to be caused by CMV reactivation [20].

The development of autoimmune disease, including autoimmune thyroiditis, type I diabetes mellitus, autoimmune hemolytic anemia, systemic lupus erythematosus, arthritis, alopecia, and vitiligo, can occur as a late complication of DIHS/DRESS (several months to years after the resolution of the condition) [22,23]. The mortality rate of DIHS/DRESS ranges from about 10% to 20% [1,15,16]. The risk of mortality in DRESS/DIHS is primarily determined by the degree of systemic involvement.

### 2.3. AGEP

AGEP mainly begins in the intertriginous areas or on the face and typically presents as numerous, small (pinhead sized, <5 mm) nonfollicular sterile pustules arising on a background of diffuse, edematous erythema, predominantly in the folds of the skin and/or on the face. Cutaneous manifestations are often accompanied by a fever of >38 °C and leukocytosis with an elevated neutrophil count. Mild eosinophilia may also be present. AGEP exhibits an acute onset, usually following drug intake. The pustules resolve within 15 days without treatment followed by pinpoint desquamation [5]. The incidence of AGEP is estimated to be 1–5 cases per million per year [5]. The EuroSCAR group conducted a multinational case-control study of 97 validated cases of AGEP and reported that a wide range of drugs have been suspected of causing these reactions, but certain medications, such as pristinamycin, ampicillin/amoxicillin, quinolones, (hydroxyl)chloroquine, sulphonamides, terbinafine, and diltiazem, are associated with a higher risk of AGEP [24]. The latter study suggested that AGEP probably has a different spectrum of causative drugs from SJS/TEN. While antibiotic agents appear to be causative drugs of both AGEP and SJS/TEN, some important triggers of SJS/TEN, such as allopurinol, antiepileptic drugs, and nevirapine, do not seem to play a major role in AGEP [24]. The mortality rate of AGEP has been reported to be about 5% [1], a relatively low rate compared with those of SJS/TEN and DIHS/DRESS.

## 3. Chemokines in SCARs

### 3.1. The Chemokine Superfamily

Chemokines are a family of small, chemoattractant proteins, which play a key role in regulating leukocyte recruitment in immunity and inflammation [6,7,25]. Chemokine function is critical for all immune cell movement, from the migration required for immune cell development and homeostasis, to that required for the generation of primary and secondary adaptive cellular and humoral immune responses or the pathological recruitment of immune cells in disease [7]. It has also been demonstrated that the chemokine system plays important roles in the priming of naïve T cells; in cell-fate decisions, such as effector and memory cell differentiation; and in regulatory T-cell function [7]. The chemokine superfamily consists of a large number of ligands and receptors. So far, over 50 endogenous chemokine ligands and 20 chemokine receptors have been identified. Chemokines are classified into four subfamilies, CC, CXC, XC, and CX3C, based on the arrangement of the two conserved cysteine residues in the N-terminal region and their activities [6,26]. The CC and CXC chemokines form the two largest groups. Besides the abovementioned structural criteria, chemokines may also be grouped according to their function; i.e., whether they are inflammatory or homeostatic [25]. Inflammatory chemokines are defined as those whose expression is upregulated under inflammatory conditions, such as in the presence of infections, inflammation, tissue injuries, or tumors, and inflammatory chemokines are mainly involved in the recruitment of leukocytes into inflamed tissues. Many inflammatory chemokines have broad target-cell selectivity and act on the cells of both the innate and adaptive immune systems [25]. Examples include chemokine (C-X-C motif) ligand 8 (CXCL8; interleukin 8 [IL-8]), CXCL9 (monokine induced by interferon gamma [IFN-γ] [MIG]), CXCL10 (IFN-inducible protein 10 [IP-10]), and chemokine (C-C motif) ligand 2 (CCL2; monocyte chemotactic protein 1 [MCP-1]) [26]. In contrast, homeostatic chemokines are those that are expressed constitutively in lymphoid or other organs and play a key role in lymphocyte migration to, and the development of, lymphoid organs. They contribute to leukocyte navigation during hematopoiesis in the bone marrow and thymus; during the initiation of adaptive immune responses in the spleen, lymph nodes (LNs), and Peyer’s patches; and in the immune surveillance of healthy peripheral tissues [25]. For instance, CCL19 (macrophage inflammatory protein-3β; MIP-3β), CCL21 (secondary lymphoid tissue chemokine; SLC), and CXCL13 are categorized as homeostatic chemokines [26]. Some chemokines span both functional categories and are referred to as dual-function chemokines [25,26]. Many dual-function chemokines are highly selective for lymphocytes and play a role in T-cell development in the thymus, as well as in T-cell recruitment to inflammatory sites [25]. Such chemokines include CCL11 (eotaxin), CCL17 (thymus and activation-regulated chemokine; TARC), CCL20 (MIP-3α), CCL22 (macrophage-derived chemokine; MDC), and chemokine (C-X3-C motif) ligand 1 (CX3CL1; fractalkine) [26].

Chemokines interact with a family of G protein-coupled receptors (GPCRs) with seven transmembrane domains that bind to extracellular ligands and transduce intracellular signals [27]. When a chemokine binds to its receptor, a calcium signaling cascade is initiated, resulting in the activation of small GTPases. This then has downstream effects, e.g., it activates integrins and actin polymerization, resulting in the development of a pseudopod, polarized cell morphology, and ultimately cell movement [6]. The chemokine receptors are also grouped into four subfamilies according to the subfamily of their major chemokine ligands: CCR, CXCR, XCR, and CX3CR [26]. Five atypical (non-chemotactic, recycling, or scavenging) chemokine receptors have also been described [26].

Chemokines are remarkably diverse. Numerous chemokines are produced simultaneously by any given tissue, and the same chemokine can be produced by diverse types of cells in response to specific signals. One of the prominent features of the chemokine system is the high level of redundancy in ligand-receptor interactions [26]. Several chemokines can act on a single receptor, and a single chemokine can bind to more than one receptor. Furthermore, multiple chemokine receptors are expressed on each cell type, and a single receptor can be expressed on several types of cells. This wide range of chemokines and chemokine receptors results in a high degree of specificity. The particular molecules expressed on a cell determine which tissue the cell will migrate into. For example, cells expressing the chemokine receptor C-C chemokine receptor 7 (CCR7) migrate to the LNs, where their ligands, CCL19 and CCL21, are expressed. It is now firmly established that chemokines play an essential role in orchestrating immune responses by coordinating the localization of immune cells in the body to generate immune responses at specific sites, and hence, the temporal and spatial expression patterns of chemokines in vivo determine the organization and functions of the cellular networks that shape the immune system [28].

### 3.2. The Role of Chemokines in CD4^+^ T-Cell Priming

**Figure 1:**
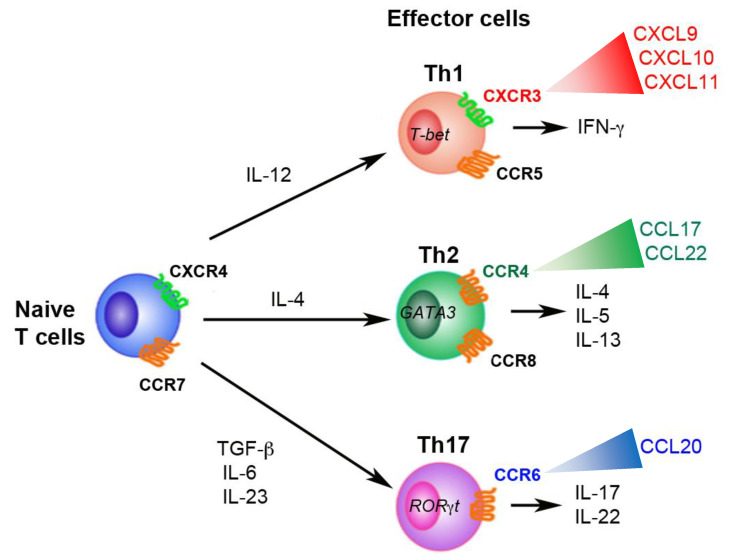
Expression of specific chemokine receptors on CD4^+^ T cells during their differentiation into effector cells. After antigen stimulation, naïve CD4^+^ T cells are activated and differentiate into distinct CD4^+^ Th-cell subsets, depending on the cytokine milieu, which drives a program of differentiation that includes the induction of the expression of key transcription factors as well as specific chemokine receptors. Polarized CD4^+^ T-cell responses may be amplified by a positive feedback loop involving chemokines. Naïve T cells differentiate into Th1 cells under the direction of the cytokine IL-12 and the transcription factor T-bet. Th1-cell-mediated inflammation is characterized by the recruitment of IFN-γ-producing CD4^+^ T cells that help protect against intracellular pathogens. The cytokine IL-4 and the transcription factor GATA3 are critical for the differentiation of naïve T cells into Th2 cells. Th2-cell-mediated inflammation is characterized by the recruitment of CD4^+^ T cells producing IL-4, IL-5, and IL-13 that help to protect against parasites and contribute to allergic responses. CD4^+^ T-cell populations that exhibit upregulated expression of the transcription factor RORγt can differentiate into Th17 cells. Th17-cell-mediated inflammation is characterized by the infiltration of CD4^+^ T cells that secrete IL-17A, IL-17F, IL-21, and IL-22. Th17 cells are considered to protect against extracellular bacteria and fungi.

After encountering an antigen, naïve CD4^+^ T cells differentiate into distinct lineages of effector T cells, depending on the cytokine milieu present in their activating environment and the induction of the expression of the transcription factors that control Th cell differentiation, which is a defining event in adaptive immunity [28] (Figure 1). Naïve T cells differentiate into Th1 cells under the direction of the cytokine IL-12 and the transcription factor T-bet. Th1-cell-mediated inflammation is characterized by the recruitment of the IFN-γ-producing CD4^+^ T cells responsible for protection against intracellular pathogens. The cytokine IL-4 and the transcription factor GATA3 play critical roles in the differentiation of naïve T cells into Th2 cells. Th2-cell-mediated inflammation is characterized by the recruitment of CD4^+^ T cells that produce IL-4, IL-5, and IL-13. These cells help to protect against parasites and contribute to allergic responses. CD4^+^ T-cell populations that exhibit upregulated expression of the transcription factor RORγt can differentiate into Th17 cells. Th17-cell-mediated inflammation is characterized by the infiltration of CD4^+^ T cells that are able to secrete IL-17A, IL-17F, IL-21, and IL-22. Th17 cells are considered to protect against extracellular bacteria and fungi. Cytokine and transcription factor-controlled Th-cell differentiation is further orchestrated within LNs by chemokines, e.g., the differentiation of CD4^+^ T cells is accompanied by the expression of particular chemokine receptors, which guides effector cells out of the lymphoid compartment and into peripheral sites of inflammation [28] (Figure 1). For instance, Th1 cells preferentially express CCR5, C-X-C motif chemokine receptor 3 (CXCR3), and CXCR6, whereas Th2 cells preferentially express CCR4 and CCR8. It is also known that Th17 cells express CCR6 [28] (Figure 1). There are considered to be two possible reasons for the expression of specific chemokine receptors during Th cell differentiation. The first is that it promotes dendritic-cell (DC)/T-cell interactions; i.e., DCs may use chemokines to facilitate encounters with antigen-specific T cells. The second possibility is that it is involved in the intranodal positioning of T cells; i.e., chemokines expressed in specific LN regions may have more global effects on the intranodal positioning of priming T cells within particular microenvironments, in order to bring these cells in contact with the antigen-presenting cells required for their differentiation [29]. The detailed chemokine systems involved in Th1- and Th2-cell differentiation are described below.

#### 3.2.1. The Chemokines Involved in Th1-Cell Priming

Early during Th1-cell differentiation, the expression of CXCR3 on antigen-specific CD4^+^ T cells is rapidly upregulated and is correlated with the ability of these cells to produce the Th1 cytokine IFN-γ. At the same time, the ligands for CXCR3, CXCL9, and CXCL10 are also rapidly expressed in the LNs [29]. The in vivo transfer of antigen-specific CD4^+^ T cells on a Cxcr3^-/-^ background into wild-type (WT) hosts that received antigen-pulsed DCs revealed that the frequency of IFN-γ-producing antigen-specific CD4^+^ T cells on a Cxcr3^-/-^ background was lower than those on a WT background, indicating that CXCR3 is required for optimal Th1-cell responses [29]. A further study involving CXCR3 ligand reporter mice demonstrated that optimal Th1-cell differentiation required CXCL9 expression by stromal cells, predominantly in the interfollicular zone, and CXCL10 expression by hematopoietic cells, predominantly in the medullary zone, which promoted the migration of CD4^+^ T cells from the T-cell zone into the interfollicular and medullary zones of the LNs [29]. These findings indicate that the interactions of CXCR3 with both CXCL9 and CXCL10 promote Th1-cell differentiation by facilitating stable contacts with DCs as well as by placing these CD4^+^ T cells into potential niches of high IFN-γ production [7,29].

#### 3.2.2. The Chemokines Involved in Th2-Cell Priming

It was also suggested that chemokine guidance optimizes Th2-cell differentiation [7]. In a murine model of contact hypersensitivity (CHS), Langerhans cells (LCs) that migrated from contact-sensitized skin into the T-cell zones of the draining LNs were found to upregulate their expression of CCL22/MDC, a CCR4 ligand, during maturation [30]. The expression of CCL22/MDC on DCs attracts activated antigen-specific CD4^+^ T cells, but not naïve or memory T cells, suggesting that the preferential recruitment of activated T cells may be a mechanism used by maturing DCs to promote encounters with antigen-specific T cells [30]. Another study of a CHS model involving CCL17-enhanced green fluorescent protein reporter mice showed that the expression of CCL17/TARC, another CCR4 ligand, in LCs was also upregulated in the LNs after antigen uptake and maturation [31]. Furthermore, they demonstrated that CCL17-deficient mice mounted impaired CHS responses, indicating that CCL17 plays a crucial role in the development of CHS responses [31].

More recently, it was demonstrated that in mice infected with *Heligmosomoides polygyrus*, the blockade of CXCL13 by either neutralizing antibodies or genetic deletion, or the genetic deletion of CXCR5 from both CD4^+^ T cells and DCs, reduced Th2 responses [32]. They demonstrated that CXCR5 expression by DCs controlled the positioning of DCs in LNs. In addition, CXCR5 expression by CD4^+^ T cells directed CD4^+^ T-cell migration out of the T-cell zone of the LNs, where *H. polygyrus*-induced Th2 responses were initiated in a CXCL13-dependent manner. They suggested that CXCR5 expression by both CD4^+^ T cells and DCs, as well as CXCL13, a ligand for CXCR5, are required for maximal Th2 responses to *H. polygyrus* [32]. Taken together, these findings suggest that both CCR4 and CXCR5 promote Th2 responses and that chemokines play an important role in optimizing the differentiation of Th2 cells.

### 3.3. Chemokines as Biomarkers of SCARs

The molecular characterization of various human diseases using gene arrays has indicated that despite the complexity of this superfamily, chemokines usually exhibit marked specificity in their associations with certain human diseases. This suggests that chemokines may be useful as biomarkers [26]. There are many inflammatory chemokines that are not normally present in the skin, and their expression can thus be a key biomarker of skin inflammation. In this section, we describe the potential roles of chemokines in SCARs, including those that we have reported previously [33,34]. SCARs, including SJS/TEN and DIHS/DRESS, can be difficult to diagnose, especially at onset, because their initial presentations are often indistinguishable from other forms of drug eruptions, and their clinical symptoms mimic those of several other diseases, such as infectious diseases [4]. It is especially challenging to diagnose DIHS/DRESS due to its diverse range of symptoms. Since SJS/TEN and DIHS/DRESS have high mortality rates, their early and accurate diagnosis are important. Therefore, diagnostic markers or predictors of these conditions have to be identified.

#### 3.3.1. Upregulation of Th2-Associated Chemokine Expression in DIHS/DRESS

**Figure 2:**
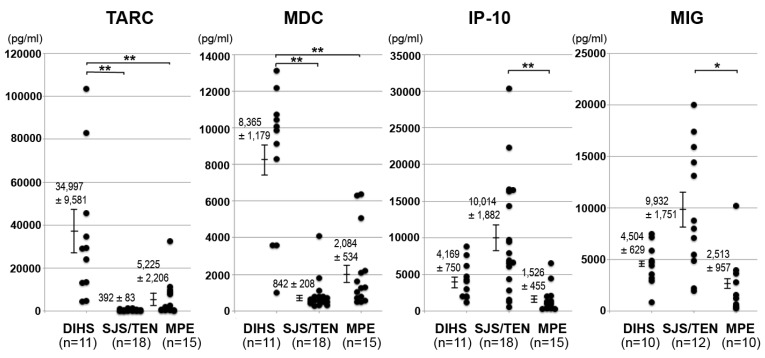
Distinct chemokine expression profiles of SCARs in the acute stage. The levels of Th2-associated chemokines (TARC and MDC) were markedly elevated in DIHS/DRESS, while Th1-associated chemokines (IP-10 and MIG) predominated in SJS/TEN. The study included 11 patients with DIHS/DRESS (7 males and 4 females; median age: 49 years, range: 16–68),18 patients with SJS/TEN (8 males and 10 females; median age: 70.5 years, range: 17–91), and 15 patients with MPE (7 males and 8 females: median age: 63 years, range: 31–78). * *p* < 0.05, ** *p* < 0.01. (adapted from [34]).

Eosinophilia [13,14] and increased plasma IL-5 levels [35] have been detected in the acute stage of DIHS/DRESS, suggesting that in this condition immune responses are polarized toward type 2 immune responses [4]. The CC chemokines CCL17/TARC and CCL22/MDC are known to be Th2-associated chemokines that bind to CCR4 on Th2 cells [7]. Therefore, we examined whether the expression of Th2-associated chemokines is upregulated in DIHS/DRESS. Indeed, DIHS/DRESS patients exhibit significantly higher serum levels of CCL17/TARC than patients with SJS/TEN or maculopapular exanthema (MPE) in the acute stage [33,34,36] (Figure 2). Serum CCL17/TARC levels increase in the acute stage of DIHS/DRESS and decrease upon remission [33,34]. This suggests that TARC may serve as a useful diagnostic marker of acute DIHS/DRESS. Double immunohistochemical staining of skin biopsy specimens from the lesional skin of DIHS/DRESS patients revealed that CD11c^+^ dermal DCs mainly expressed CCL17/TARC, suggesting that CD11c^+^ dermal DCs are the major source of this chemokine [33]. We also found that the serum TARC levels of DIHS/DRESS patients with HHV-6 reactivation were significantly higher than those of DIHS/DRESS patients without HHV-6 reactivation, indicating that serum CCL17/TARC levels are associated with HHV-6 reactivation [36]. Another study indicated that in DIHS/DRESS serum CCL17/TARC levels were correlated with the severity of skin and mucosal lesions, fevers, and liver and kidney dysfunction; HHV-6 and CMV DNA copy numbers; and serum IL-5, IL-10, and soluble IL-2 receptor levels [33,37], suggesting that the serum CCL17/TARC level could be used as a marker of the clinical and immunological status of DIHS/DRESS patients [37]. We also reported the case of a patient with DIHS/DRESS who developed frequent flare-ups during the course of the disease, and their serum CCL17/TARC levels increased in parallel with the flare-ups of clinical symptoms, particularly skin eruptions [38]. The findings of this case suggest that serum CCL17/TARC levels could also be useful as an indicator of relapses in DIHS/DRESS. In addition, serum CCL17/TARC levels can be assessed repeatedly during treatment monitoring to determine when corticosteroid doses should be tapered [38]. Taken together, serum CCL17/TARC levels are a useful indicator of both the onset and progression of DIHS/DRESS and can be used for its early diagnosis and evaluation. However, it should be acknowledged that elevated serum CCL17/TARC levels are not specific to DIHS/DRESS and can also be found in many other skin diseases, such as atopic dermatitis (AD) [39], bullous pemphigoid (BP) [40], and mycosis fungoides (MF) [41]. However, it should be emphasized that the serum CCL17/TARC levels of DIHS/DRESS patients are far higher than those of patients with AD, BP, or MF. A series of studies conducted by Kakinuma et al. demonstrated that the mean serum CCL17/TARC levels (normal value: <450 pg/mL) of AD, BP, and MF patients were 2338.70 ± 302.83, 1151.5 ± 885.6, and 2889.6 ± 725.5 pg/mL, respectively [39,40,41]. On the other hand, in three of our studies the mean serum CCL17/TARC levels of DIHS/DRESS patients were found to be 31,259.63 ± 6374.92 [33], 34,997 ± 9581 [34], and 21,023 ± 17,040 pg/mL [36], respectively, demonstrating that the serum CCL17/TARC levels of patients with DIHS/DRESS are >10-fold greater than those of patients with other skin diseases, such as AD, BP, and MF. A clinical trial to validate the effectiveness of using serum CCL17/TARC levels for distinguishing DIHS/DRESS from other types of drug eruptions is currently ongoing in Japan [4].

We also found that the serum levels of CCL22/MDC, a chemokine related to CCL17/TARC, were markedly higher in DIHS/DRESS than in other forms of drug eruptions during the acute stage [34] (Figure 2). The levels of MDC declined upon remission [34].

The elevated levels of the Th2-associated chemokines CCL17/TARC and CCL22/MDC seen in patients with DIHS/DRESS in the acute stage also support the predominance of type 2 immune responses in DIHS/DRESS. Significantly higher proportions of circulating IL-4- and IL-13-producing CD4^+^ T cells (Th2 cells) were seen in patients with DIHS/DRESS [42], and CD134 (OX40) expression on CD4^+^ T cells and OX40L expression on peripheral blood mononuclear cells (PBMCs) were upregulated in the acute stage of DIHS/DRESS [43,44], which are also indicative of type 2 skewing because OX40-OX40L ligation is known to promote Th2-cell differentiation [45].

#### 3.3.2. CXCL10/IP-10 as a Biomarker of Late Sequelae in DIHS/DRESS

The outcomes of DIHS/DRESS are often unpredictable. Some patients with DIHS/DRESS develop various autoimmune sequelae after the resolution of the disease [4]. However, it is currently unknown why some DIHS/DRESS patients develop autoimmune diseases, and there are no reliable markers for predicting these outcomes. In a recent study by Yang et al., significantly higher plasma CXCL10/IP-10 levels were detected in the acute stage of DIHS/DRESS in patients with long-term sequelae, such as Hashimoto’s thyroiditis and fulminant type 1 diabetes mellitus, than in those without long-term sequelae or those with HHV-6 reactivation [46]. This suggests that higher plasma CXCL10/IP-10 levels are associated with the development of long-term sequelae and could be a useful biomarker for predicting the development of long-term sequelae in patients with DIHS/DRESS at an early stage [46]. CXCL10/IP-10 and its receptor, CXCR3, appear to contribute to the pathogeneses of many autoimmune diseases, as the serum and/or tissue expression levels of CXCL10/IP-10 have been reported to be increased in both organ-specific autoimmune diseases, including autoimmune thyroiditis, Graves’ disease, and type I diabetes, and systemic autoimmune diseases, including rheumatoid arthritis, systemic lupus erythematosus, and systemic sclerosis [47].

#### 3.3.3. Upregulation of Th1-Associated Chemokine Expression in SJS/TEN

In contrast to the elevated levels of the Th2-associated chemokines CCL17/TARC and CCL22/MDC seen in DIHS/DRESS, we found that the expression levels of the Th1-associated chemokines CXCL9/MIG and CXCL10/IP-10 were higher in SJS/TEN than in MPE [34] (Figure 2). CXCL9/MIG and CXCL10/IP-10 are related chemokines of the CXC subfamily that are known to share the CXCR3 receptor [7]. These results were consistent with the findings of previous studies in which SJS/TEN was characterized by a predominantly Th1 activation pattern [48,49].

Since the expression of Th2-associated chemokines is markedly upregulated in DIHS/DRESS, while Th1-associated chemokines are dominant in SJS/TEN, TARC/MDC and IP-10/MIG expression profiles can be used to aid the differential diagnosis of SCARs [34].

## 4. Viral Chemokines in SCARs

### 4.1. Viral Subversion of the Immune System Through Chemokines

Close associations are known to exist between viruses and chemokines. For instance, the two main chemokine receptors, CXCR4 and CCR5, function as portals for the entry of human immunodeficiency virus 1 (HIV-1) [6]. Viruses also produce chemokine system mimics to subvert immune responses. Many chemokine- or chemokine receptor-like molecules have been identified with the sequencing of various viral genomes [6]. At least three different chemokine-based strategies are known to be used by viruses to subvert host immune responses [6]. The first strategy is the use of chemokine homologs, which have probably been copied by viruses and modified to induce broad antagonistic activity. Second, viruses produce membrane-expressed chemokine receptor homologs that soak up chemokines to dampen host responses. The third strategy involves the use of secreted chemokine-binding proteins that bind to chemokines with high affinity although they do not resemble any other proteins. Many viruses appear to widely use chemokine-based strategies for immune subversion, emphasizing the importance of both chemokines and cell recruitment for antiviral responses. A remarkable feature of most viral chemokines or chemokine-binding proteins is their broad chemokine or receptor-binding capabilities, which suggests that viruses need to circumvent chemokine redundancy to ensure effective immune subversion [6].

### 4.2. Chemokine Mimicry in HHV-6 Reactivation

It is now widely accepted that DIHS/DRESS can be associated with the reactivation of HHV-6, as HHV-6 reactivation is one of the diagnostic criteria for DIHS [13] although some investigators still dispute the role of HHV-6 reactivation in DIHS/DRESS [4]. HHV-6 DNA is detected during flare-ups of symptoms, indicating the relevance of HHV-6 reactivation to the flaring-up of clinical symptoms in DIHS/DRESS [50]. The finding that HHV-6-derived microRNAs have been detected in the sera and PBMCs of patients with DIHS/DRESS also supports this [51]. Furthermore, it has been reported that HHV-6 DNA was detected in the skin [18], LNs [52], and kidneys [53] of DIHS/DRESS patients, suggesting that rashes, lymphadenopathy, and renal failure might be closely related to HHV-6 reactivation in DIHS/DRESS. Thus, HHV-6 reactivation might be associated with the pathogeneses of various clinical symptoms, such as organ failure, rashes, and lymphadenopathy, in patients with DIHS/DRESS [4].

As is the case with many other viruses, HHV-6 also uses chemokine-related molecules for immune subversion.

HHV-6 has three chemokine system mimics: two are receptors (CCRs), and one is a chemokine agonist (a CC chemokine) [54]. As these chemokine system mimics might be important for HHV-6 transmission and HHV-6 pathogenicity in DIHS/DRESS, the two GPCR homologs, U12 and U51, and the chemokine homolog U83 are described in detail below. Another possible role of chemokine system mimics is that elevated levels of certain chemokines, such as CCL17/TARC and CCL22/MDC [33,34,36], might directly activate HHV-6 through the chemokine receptor homologs of HHV-6 although this needs further study.

#### 4.2.1. U12

DNA sequence alignment has identified two candidate 7-transmembrane GPCRs, open reading frames (ORFs) U12 and U51, within the HHV-6 genome [55]. Another study demonstrated that the U12 gene of HHV-6B functionally encodes a chemokine receptor for β-chemokines, including regulated on activation, normal T-cell expressed and secreted (RANTES), MIP-1α and MIP-1β, and MCP-1, and is capable of transducing specific chemokine signals that lead to the transient elevation of the intracellular Ca^2+^ concentration (this occurs most potently in response to RANTES) to the cytoplasm [56]. However, the biological roles of the viral chemokine receptors produced by HHV-6 are not yet known. It is suggested that through the actions of these receptors, the virus is able to regulate cellular processes to enhance its replication or inhibit an apoptotic response, thereby allowing the establishment of a latent infection. It is also suggested that in latently or persistently infected cells viral chemokine receptors are activated by chemokines, resulting in reactivation of the virus, since U12 is a late gene. Alternatively, viral chemokine receptors may act as molecular mimics to divert chemokines from their natural ligands and thereby subvert local immune responses [56].

#### 4.2.2. U51

The U51 gene is one of the two GPCRs possessed by HHV-6 [55]. U51 binds to multiple CC chemokines, despite having exceptionally low sequence similarity to mammalian and other viral chemokine receptors (<23% amino acid identity) [54,57]. It exhibits specific binding to RANTES and competitive binding with other β-chemokines, such as eotaxin; MCP-1, -3, and -4; as well as the HHV-8 chemokine vMIPII [57]. In epithelial cells that were secreting RANTES, U51 expression resulted in specific downregulation of RANTES transcription [57]. The suppression of RANTES expression may affect the recruitment of inflammatory cells that the virus can infect or modulate a protective inflammatory response to aid the spread of the virus; therefore, U51 may function as an immune evasion molecule [57]. Such mimicry of host receptors by viral proteins, leading to the downregulation of chemokine expression, is an immunomodulatory mechanism that could facilitate viral spread from initial sites of infection in epithelia [54,57].

Although U51 has been reported to bind to certain CC chemokines, such as RANTES, no signaling activity has been found to be associated with this interaction, and the biological function of U51 remains uncertain [58]. Using short interfering RNAs specific to U51, Zhen et al. demonstrated that U51 is a positive regulator of HHV-6 replication, probably by promoting membrane fusion and facilitating the cell-cell read of HHV-6 [58].

#### 4.2.3. U83

DNA sequence analysis of HHV-6 revealed that the putative protein encoded by an ORF of the U83 gene in HHV-6 variant B resembled a human chemokine [55]. Using the purified U83 protein, Zou et al. demonstrated that this chemokine homolog of HHV-6B was capable of inducing transient calcium mobilization in THP-1 cells and efficient migration of such cells, indicating that U83 is a functional chemokine [59]. It is known that HHV-6 predominantly replicates in CD4^+^ T cells in vivo and in vitro [60,61] and may establish latent infections in monocyte/macrophage-lineage cells [62]. Zou et al. suggested that the U83 protein may play an important role in HHV-6 propagation in vivo by activating and recruiting mononuclear cells to sites of viral replication, thus aiding the spread of HHV-6B [59].

A study by Clark et al. demonstrated that the U83 produced by HHV-6B (U83B) is monospecific to CCR2 [63]. U83B-N-terminal 17-mer peptides can activate and induce chemotaxis in CCR2^+^CD14^+^CD16^+^ monocytes [63]. HHV-6B U83B, like the human chemokine CCL2 (MCP-1), is monospecific to CCR2, a chemokine receptor expressed on monocytes. Taken together, U83 induces chemoattraction to facilitate latent infections and dissemination in monocytes, and it also competes with CCL2 to activate chemokine receptors, thereby diverting the host’s cellular responses.

## 5. Conclusions

Given the overall rarity, but high morbidity and mortality rates of SCARs, disease-related biomarkers of these conditions are desired. Ideally, such biomarkers should be minimally invasive, biologically relevant, and linked to disease mechanisms. As DIHS/DRESS exhibits Th2 skewing, the serum CCL17/TARC level is one of the most promising biomarkers of DIHS/DRESS, and its utility is currently being confirmed in Japan. In comparison with other skin diseases in which serum CCL17/TARC levels are known to be elevated, the serum levels of CCL17/TARC seen in DIHS/DRESS are extremely high and are correlated with clinical severity and prognosis. Other potential biomarkers of SCARs include other Th2-associated chemokines, such as CCL22/MDC for DIHS/DRESS and the Th1-associated chemokines CXCL9/MIG and CXCL10/IP-10 for SJS/TEN. Large-scale clinical trials evaluating their utility are required. It is also suggested that the roles of chemokines in the control of inflammatory/immune responses and cell migration make them compelling targets for drug development.
